# De Garengeot Hernia: A Case Report of an Incidental Finding

**DOI:** 10.7759/cureus.73817

**Published:** 2024-11-16

**Authors:** Simrat Tiwana, Syed A Kabir

**Affiliations:** 1 Surgery, Walsall Manor Hospital, Walsall, GBR; 2 General and Bariatric Surgery, Walsall Manor Hospital, Walsall, GBR

**Keywords:** appendix, de garengeot, femoral, hernia, laparoscopic

## Abstract

De Garengeot hernia is a rare occurrence characterised by the presence of the appendix within a femoral hernia. This type of hernia is notable for its rare anatomical presentation. In rare instances, the appendix can present as inflamed or necrotic in which case it may present as an emergency. In many instances, De Garengeot hernia is discovered incidentally during surgical repair of a hernia. This incidental finding raises an important consideration for surgeons. This study reports a case of De Garengeot hernia identified as an incidental intraoperative finding. It aims to enhance awareness of the condition, ultimately improving patient outcomes and management.

## Introduction

Femoral hernias account for approximately 2-4% of groin hernias [1]. The incidence of an appendix in a femoral hernia is reported in 0.5-5% of femoral hernias and predominantly on the right side [2,3]. In 0.08-0.13% of cases, the appendix can present as inflamed or necrotic in which case it may present as an emergency [4]. We report the presentation of a patient with a reducible right-sided femoral hernia with a vermiform appendix hernia.

## Case presentation

**Figure 1:**
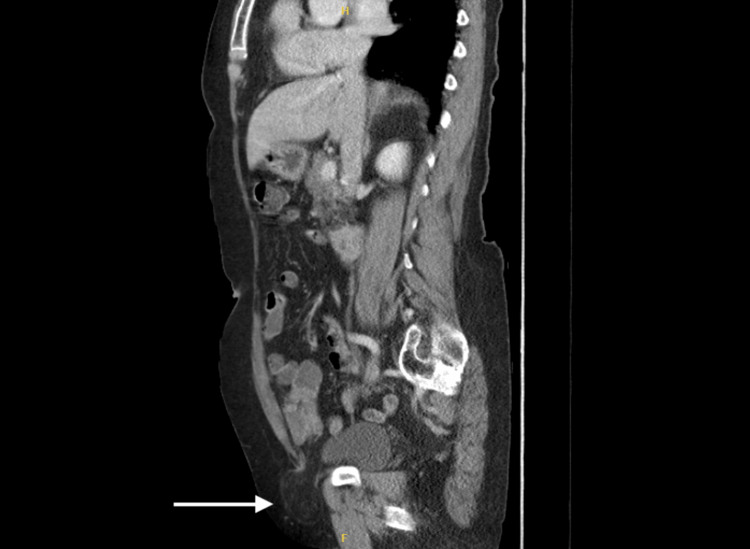
A computed tomography scan demonstrating a right-sided hernia (white arrow).

An 85-year-old woman with a suspected right-sided femoral hernia presented for elective surgery. The patient initially reported a painful swelling in the groin. No other symptoms such as abdominal pain, nausea, vomiting or change in bowel habits were observed. Clinical examination revealed a reducible lump in the right groin, which was suspected to be a femoral hernia. An ultrasound scan confirmed a direct hernia containing fat and bowel. Blood tests showed no signs of infection with the levels of white blood count and C-reactive protein (CRP) test being within normal limits. A computed tomography (CT) done prior to surgery can be seen in Figure 1. This demonstrates a sagittal view of a right-sided likely femoral hernia but does not illustrate herniation of the appendix, which was only discovered intraoperatively.

The patient underwent a laparoscopic repair of the femoral hernia. A 10 mm umbilical port was placed alongside two 5 mm ports in the right and left upper quadrants. This allowed for the creation of a pneumoperitoneum. Intraoperatively, a femoral hernia containing an appendix in the sac was found. The base of the appendix and mesentery were separated and fatty tissue from the femoral hernia was reduced. The appendix was not inflamed and was reduced alongside the fatty tissue. The hernia defect was closed using an absorbable mesh plug. The patient recovered without any postoperative complications and was discharged on the same day as her surgery.

## Discussion

Herniation of the appendix within a femoral hernia was first described in 1731 by Rene Jacques de Garengeot (De Garengeot hernia) [5]. This condition has primarily been reported in case reports. A literature search conducted in 2022 analysing published papers in the English language revealed 127 reported cases between 1898 and 2019 [6]. The true incidence is difficult to calculate. As such, analysis regarding the diagnosis and the surgical management of such patients is limited.

De Garengeot hernia presents clinically as a non-reducible groin mass that is tender to palpate [7]. Symptoms of obstruction, such as abdominal pain, vomiting or fever, are not usually reported [8]. This is highlighted in the presentation of our case that simply presented as a reducible groin mass. Preoperative tests may reveal leukocytosis and elevated CRP; however, these laboratory findings were not observed in the present case. There is a predisposition for females with a female-to-male incidence ratio of 5:1 within the age range of 36 to 92 years [1,9].

Preoperative diagnosis is rare, with most reported cases diagnosed intraoperatively [10]. Computed tomography and sonography have been successfully used [11]. Imaging of the De Garengeot hernia will reveal an appendix extending into the femoral hernia. If inflamed, the appendix presents with mural thickening and surrounding fat stranding. While imaging studies can be utilised in the evaluation of a possible hernia, diagnosis is often based on clinical assessment with imaging used as an aid for confirmation.

Due to the rarity of this finding, there is no standard surgical approach established for this condition [12]. Based on the clinical presentation, surgery can be performed either open or laparoscopically [13]. In this case, due to a lack of comorbidities, a laparoscopic approach was decided. Treatment options include simple hernia repair or the use of mesh, with or without appendectomy.

The definitive treatment of De Garengeot hernia involves surgical appendectomy and hernia repair together [14]. However, it is suggested that if the appendix is normal, appendectomy may not be necessary [15]. This was the case for our patient, with closure being achieved with a mesh plug. Due to limited data, the recurrence rate of De Garengeot hernia following this approach remains undetermined [6].

Although the incidence of De Garengeot hernia is low, it is important to remain aware of it. The approach to treatment is best decided by clinical presentation and intraoperative findings.

## Conclusions

De Garengeot hernia is a rare presentation describing the presence of the appendix within a femoral hernia. During our literature review, we have come across cases of De Garengeot hernia that have been diagnosed preoperatively with imaging. While imaging techniques like CT and ultrasound assist in diagnosis, most cases of De Garengeot hernia are diagnosed intraoperatively. We present this case as a careful consideration for surgeons to remain mindful of the possibility of herniation of the appendix into a femoral hernia, as this can impact the surgical approach taken for repair. Surgical management can be laparoscopic or open, with definitive treatment involving hernia repair and, if indicated, an appendectomy.
